# Stage 1 Registered Report:  Rationale, design and study protocol for a prospective, exploratory study Predicting Response to image-Guided Cryoneurolysis of the Greater Occipital Nerve (CryoGON) in Chronic Migraine Using a Conventional Greater Occipital Nerve Block.

**DOI:** 10.12688/f1000research.171975.2

**Published:** 2026-02-04

**Authors:** Tore Wergeland, Melanie Rae Simpson, Irina Aschehoug, Lise Rystad Øie, Kristina Devik, Martha Brakestad Larsen, Sverre Myren, Erling Andreas Tronvik, Daniel Fossum Bratbak

**Affiliations:** 1 Norwegian Centre for Headache Research (NorHEAD), Norwegian University of Science and Technology (NTNU), Trondheim, Norway; 2 Department of neurology and neurophysiology, St Olav’s University Hospital, Trondheim, Norway; 3 Department of Neuromedicine and Movement Sciences, Norwegian University of Science and Technology, Trondheim, Norway; 4 Department of Public Health and Nursing, Norwegian University of Science and Technology, Trondheim, Norway; 5 Department of Neurology, Nord Trøndelag Trust, Namsos, Norway; 6 Department of Neurology, Molde Hospital, Molde, Norway; 7 Department of Neurology, Ålesund Hospital, Ålesund, Norway; 8 Department of Health Sciences in Ålesund, Norwegian University of Science and Technology, Ålesund, Norway; 9 Department of Neurosurgery, St Olav's University Hospital, Trondheim, Norway

**Keywords:** chronic migraine, Cryoneurolysis, GON

## Abstract

**Background:**

Chronic migraine is frequently refractory to available preventive therapies. Image-guided cryoneurolysis of the greater occipital nerve (CryoGON) may provide longer-lasting relief than conventional greater occipital nerve (GON) blocks. This Stage 1 Registered Report describes a prospective, exploratory study evaluating whether response to a conventional GON block predicts subsequent response to CryoGON, and assesses open-label safety, tolerability, feasibility and efficacy of CryoGON.

**Methods:**

The study is planned as a single-site, investigator-initiated, open-label clinical investigation. Adults with chronic migraine will complete a ≥28-day electronic baseline diary before receiving bilateral conventional GON blocks, followed by a ≥28-day observation. Eligible participants then undergo navigation-guided CryoGON with 12-week follow-up. The primary endpoint is the association between change in moderate-to-severe headache days after GON block (weeks 1–4) and after CryoGON (weeks 9–12). Key secondary endpoints estimate the diagnostic performance of GON-block response applying a ≥30% reduction threshold for treatment effect to identify CryoGON responders, including sensitivity, specificity and predictive values. Additional endpoints include change in migraine days, patient-reported global impression of change, Migraine-Specific Quality of Life Questionnaire v2.1, procedure tolerability, technical success, and safety (treatment-emergent adverse events and adverse device effects). Women of childbearing potential, including pregnant and breastfeeding participants, are eligible for participation.

**Discussion:**

This study will provide the first systematic evaluation of whether a simple, widely available GON block can guide patient selection for CryoGON and will generate foundational open-label data on its clinical performance and safety. Findings will inform the design and justification of a subsequent sham-controlled trial.

**Trial registration and approvals:**

The study will preregister at ClinicalTrials.gov. and obtain all necessary ethical approvals before study initiation.

Sponsor: St. Olav’s University Hospital, Trondheim, Norway.

Funding: Stiftelsen Dam (grant SDAM_FOR701626).

## Introduction

Migraine is a disabling primary headache disorder with a lifetime prevalence of 17% of the population and ranking as the second leading cause of disability in females under 50 years of age. ^1^ Chronic migraine, defined by ≥15 headache days per month for >3 months with ≥8 migraine days, affects 1.4–2.2% of the population and imposes a major personal and socioeconomic burden. ^2^ Current preventive treatments include repurposed oral drugs and botulinum toxin A, as well as treatments targeting the CGRP neuropeptide system, yet many patients remain insufficiently treated. Suitable treatment options for woman of childbearing potential not using highly effective contraception, pregnant and nursing woman are even more limited. Indeed, chronic migraine and related complications, such as medication overuse headache causes women of childbearing potential (WOCBP) to avoid childbearing or to having fewer children then intended, ^3^ underscoring the need for novel non-pharmacological treatment strategies.

Greater occipital nerve (GON) block with a local anaesthetic is widely used as a transitional or rescue therapy in migraine and trigemino-autonomic cephalalgias. ^4^ A recent randomised trial and a meta-analysis support modest efficacy in chronic migraine, with reductions in headache days and responder rates superior to placebo. ^5^ However, short duration of effect and the need for repeated injections limit its preventive utility.

**Figure 1.**
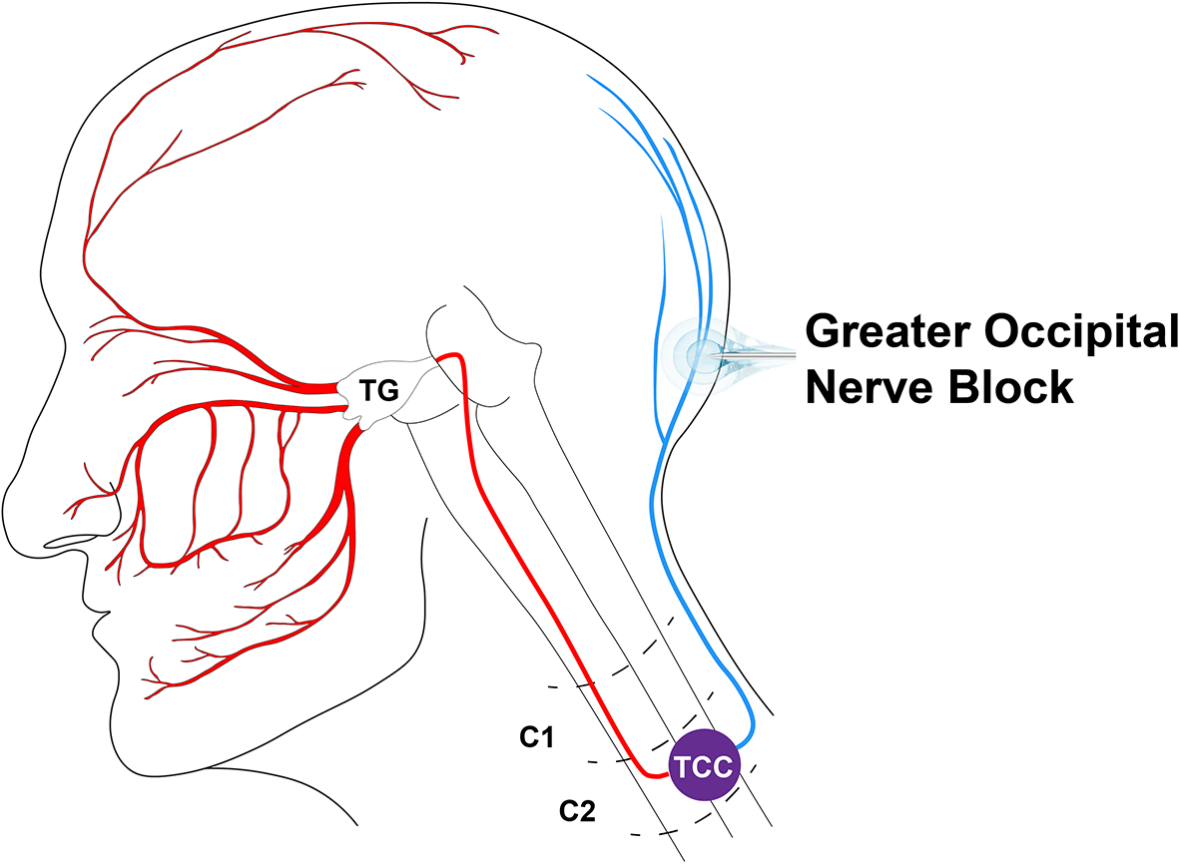
Lateral view of the left side of the head illustrating the trigeminal (red) and occipital (blue) nerves. Together, these nerves provide sensory innervation to the entire head and are functionally coupled within the trigeminocervical complex (TCC). A needle illustration indicates the approximate segment of the greater occipital nerve targeted by conventional GON blocks and by the CryoGON procedure. Bilateral treatment (not shown) is performed for both interventions.

The analgesic effects of GON blocks on headache disorders, often characterized by pain in trigeminal regions, suggest a functional interaction between the occipital and trigeminal systems. The functional overlap between occipital and trigeminal sensory processing takes place at the level of the upper cervical spinal cord where nociceptive fibres from both regions converge on the trigeminocervical complex ( Figure 1). This functional and anatomical coupling is demonstrated in several animal studies. ^6– 10^ In humans, when the greater occipital nerve (GON) is pharmacologically blocked in participants without a headache diagnosis or other pain condition, a reduced nociceptive trigeminal response was observed compared to placebo. ^11^ Similar studies have not been performed in headache sufferers.

Cryoneurolysis is an established treatment in pain medicine. ^12^ By applying cold temperatures to neural structures, it induces reversible Wallerian degeneration (axonotmesis) with subsequent regeneration, resulting in a long-acting but reversible nerve block. Cryoneurolysis has demonstrated efficacy in trigeminal and occipital neuralgia, with reported pain relief lasting several months. ^13– 16^ A sham-controlled study in cervicogenic headache, in which the control-arm received GON-block with steroids, did not demonstrate a statistically significant between-group effect. The investigators in this trial used anatomical landmarks and nerve stimulation to guide the cryoprobe, and insufficient targeting could have reduced the efficacy in the cryoneurolysis arm. It is to date no published reports on efficacy or safety in primary headache conditions to our knowledge, but cryoneurolysis towards the GON is currently studied in an open label pilot study in chronic migraine (NCT06554886).

Surgical navigation systems enable accurate percutaneous targeting of deep neural structures using pre-procedural imaging and real-time tracking. Applied to the GON, navigation-guided cryoneurolysis (CryoGON) offers a minimally invasive, office-based procedure with the potential to provide longer-lasting preventive benefit than conventional GON blocks.

This trial is designed to determine whether response to a conventional GON block predicts subsequent response to CryoGON in chronic migraine, thereby assessing the clinical utility of the block as a decision tool. Secondary objectives include evaluation of open-label efficacy, safety, tolerability, and feasibility of the CryoGON procedure. To that end, this trial will inform the design of future studies by assessing the feasibility and rational for conducting a sham-controlled trial.

### Study objectives

Primary objective

To evaluate whether clinical response to a conventional greater occipital nerve (GON) block is associated with treatment response to navigation-guided cryoneurolysis of the GON (CryoGON) in patients with chronic migraine.

Secondary objective

To evaluate the open label clinical performance of CryoGON as a potential new treatment for chronic migraine.

### Timeline

The study is planned to be initiated in 2026 with a duration of recruitment for 2 years ending in 2028. Data lock is thus planned in 2028 and submission of the stage 2 manuscript by end of 2028 or start of 2029. Publication will adhere to the Vancouver guidelines. We do not intend to use professional writers.

## Methods

This protocol publication is based on the clinical investigation plan (study protocol), version 1.0, dated 14th of October 2025. This will be a clinical investigation of a medical device for cryoneurolysis, that will be conducted in accordance with the Declaration of Helsinki, Norwegian law (including the Medical Device Regulation), and ISO 14155:2020 (Clinical investigation of medical devices for human subjects – Good clinical practice). The trial will be initiated following approval by the competent authorities, the Regional Committee for Medical and Health Research Ethics and institutional approval by the sponsor. The inclusion and data collection of each study participant will commence only after their written informed consent has been obtained. Amendments to the protocol will be implemented only after appropriate review and approval by the relevant parties. The study will be prospectively registered at ClinicalTrials.gov prior to enrolment of the first participant. All items from the WHO Trial Registration Data Set will be included in the ClinicalTrials.gov record and correspond to the information provided in this protocol publication.

The study is investigator-initiated and will be sponsored by a public hospital (St Olav’s university hospital, 7006 Trondheim, Norway, post.nevro@stolav.no). It will be conducted under the auspices of the Norwegian Headache Research Center (NorHead), a publicly funded academic center at the Norwegian University of Science and Technology. Funding for this study will be provided by the DAM Foundation (Stiftelsen Dam, grant no SDAM_FOR701626), a Norwegian non-profit foundation that allocates research grants from the surplus of the national lottery operator (Norsk Tipping). The grant application was submitted in collaboration with the national patient organization *Hodepine Norge (eng. Headache Norway)*, which also coordinates patient involvement in the design and conduct of the study. A prerequisite of the DAM Foundation grant award is that dissemination follows the two-stage Registered Report publication format.

The funding body, sponsor or any other party than the authors of this paper, had no role in the study design, in writing the clinical investigation plan (study protocol) and the decision to submit the protocol for publication.

Participants are insured through the Norwegian System of Patient Injury Compensation (Norsk pasientskadeerstatning). No post-trial care is provided with-in the framework of the trial.

### Study design

The trial will be conducted as a single-site, open-label study within the Norwegian public healthcare system, which reimburses patient travel and accommodation expenses. Should any co-payment be required, these costs will be covered by the study. No remuneration or financial incentives will be offered beyond reimbursement of necessary expenses. The study will be designed and conducted according to the guidelines of the International Headache Society. ^17^

Study recruitment

Recruitment will be facilitated through the NorHead national research infrastructure. Once enrolment opens, study information will be made available on governmental and institutional websites as well as through social media platforms. Patients may be referred by their treating physician or may self-register via the NorHead online portal. Study personnel will perform pre-screening of candidates, and only patients considered potentially eligible will be scheduled for a formal eligibility assessment by a neurologist.

Eligibility criteria

Inclusion and exclusion criteria are summarized in Table 1. In brief, eligible participants will be adults (≥18 years) with chronic migraine, with or without aura, fulfilling the International Classification of Headache Disorders (ICHD-3) criteria and verified by a neurologist at inclusion. Participants must have a history of chronic migraine for at least one year, with onset of episodic migraine before age 50 and onset of chronic migraine before age 65. Preventive medications must have been stable for at least three months (or ≥5 half-lives, whichever is longer) prior to screening, and participants must agree to maintain their regimen throughout the study. Individuals treated with onabotulinumtoxinA must have received at least three consecutive PREEMPT-protocol injection cycles with stable intervals. Only patients deemed suitable for both a conventional GON block and CryoGON by the investigator, and capable of providing written informed consent, will be enrolled.

**Table 1.** inclusion and exclusion criteria.

Inclusion criteria	Exclusion criteria
1. Participant must be 18 years at the time of signing the informed consent. 2. Migraine, with or without aura, fulfilling the International Classification of Headache Disorders (ICHD) III criteria 1.3. for chronic migraine at time of inclusion, verified by a neurologist. 3. Chronic migraine at least for a period of 1 year prior to inclusion. 4. Debut of episodic migraine before the age of 50, and chronic migraine before the age of 65. 5. Subject has had no change in type, dosage or dose frequency of preventive headache medications <3 months prior to inclusion, or a minimum of 5 half-lives, whichever is longer. In the case of treatment with botulinumtoxin A following the PREEMPT injection regimen, a minimum of three consecutive treatments with equal between-treatment intervals are required. 6. Subject agrees to maintain current preventive headache medication regimens (no change in type, frequency, or dose) during baseline until 12 weeks following CryoGON-treatment. 7. Be an appropriate candidate for the study interventions required in this study on the basis of the clinical judgment of the investigator. 8. Capable of giving signed informed consent which includes compliance with the requirements and restrictions listed in the informed consent form (ICF) and in the study protocol.	1. Subject been treated with injections targeting the greater occipital nerve or the Occipital/suboccipital region, except for onabotulinumtoxin treatment in accordance to the PREEMT injection regime the last 12 weeks prior to baseline, or during the baseline period. 2. Subject is unable to differentiate a migraine day from other concomitant headache disorders. 3. Subject with secondary headache conditions, with the exception of medication overuse headache. 4. Subject with occipital neuralgia or neuropathy, or that otherwise exhibit disturbed sensory function in the innervation area of the GON that in the opinion of the investigator can disturb the evaluation of CryoGON procedure success. 5. Subject has had a change in type, dosage or dose frequency of preventive headache medications during the baseline period. 6. Subject is or has been treated with occipital nerve stimulation. 7. Ongoing abuse of drugs (including narcotics) or alcohol. 8. More than 4 days of opioid use per month (including codeine and tramadol), and any use of barbiturates. 9. Other pain conditions, not intended to be treated in this study, that in the opinion of the investigator could interfere with study procedures, accurate pain reporting, and/or confound evaluation of study endpoints. 10. High probability of deterioration due to other medical conditions, that in the opinion of the investigator may confound outcome assessment. 11. Patients with comorbid psychiatric disorders with psychotic or other symptoms making compliance with the study protocol difficult, at the discretion of the investigator. 12. Other coexisting current medical conditions, including, but not limited to, bleeding diathesis, cryopathies (cryoglobulinemia, paroxysmal cold hemoplobinuria), and thrombophilia, that presents excess procedural risk, in the opinion of the investigator. 13. Abnormal pain behavior, inappropriate medication use and/or unresolved psychiatric illness, that in the opinion of the investigator are significant enough to impact perception of pain, compliance with intervention and/or ability to evaluate treatment outcome. 14. Anomaly or trauma which renders the planned procedure difficult. 15. Subject currently has an active abscess or a local infection at the site of intervention based on present symptoms. 16. Subject has been diagnosed with any major infectious processes such as osteomyelitis, or primary or secondary malignancies involving skull or site of intervention that have been active or required treatment in the past 6 months. 17. Current participation in another treatment study.

Key exclusion criteria include prior GON-targeted injections within 12 weeks of baseline, inability to differentiate migraine days from other headache types, or coexisting secondary headache disorders (except medication overuse headache). Patients with occipital neuralgia or neuropathy or other pain conditions that may confound evaluation of study outcomes will be excluded. Additional exclusions include unstable preventive medication regimens, prior or current occipital nerve stimulation, frequent opioid or any barbiturate use, significant psychiatric comorbidity or abnormal pain behaviour, relevant medical conditions increasing procedural risk (e.g. bleeding diathesis), and ongoing treatment site infection.

Study outline

**Figure 2.**
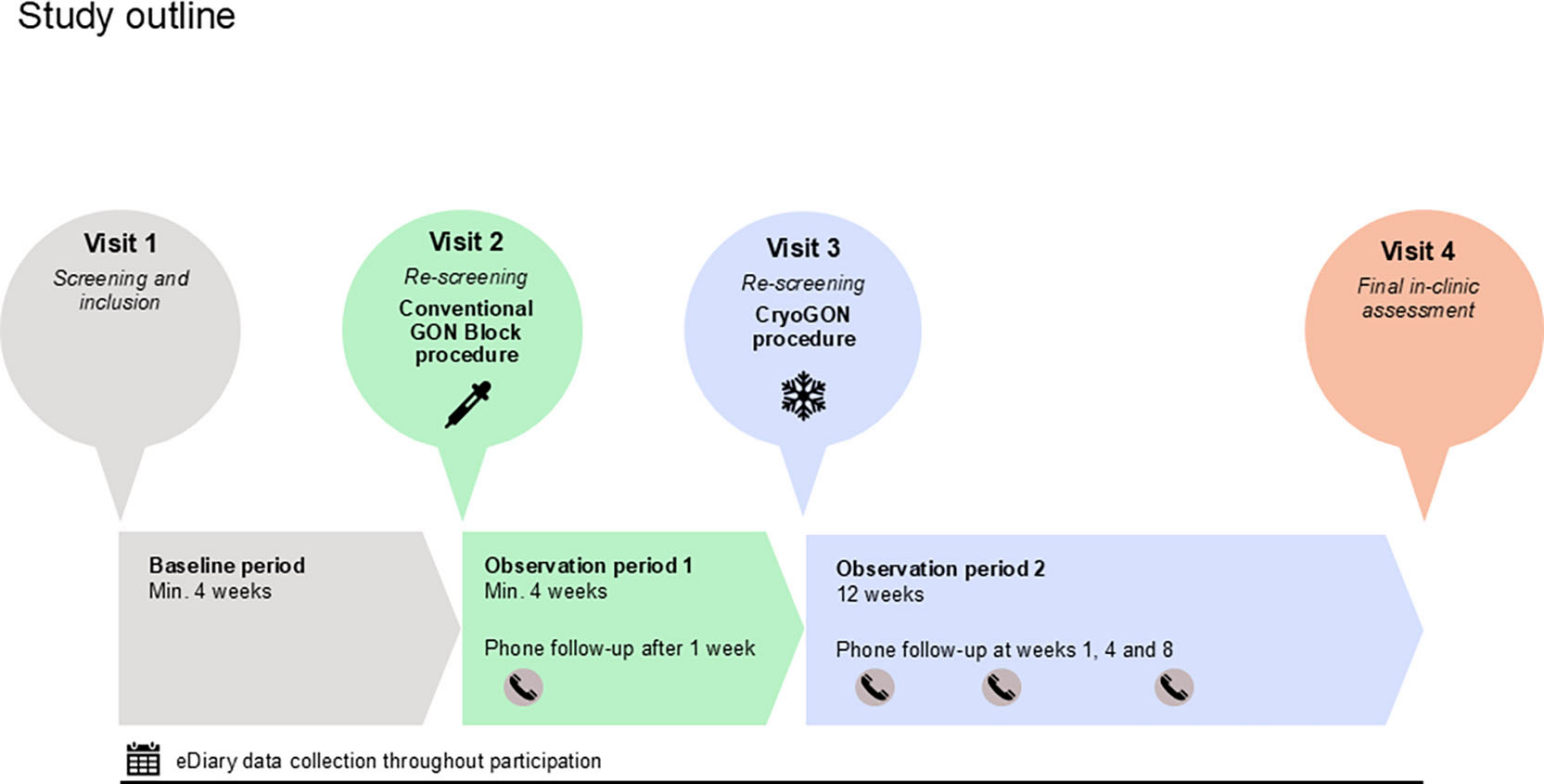
Study outline. The study includes four in-clinic visits separated by observation periods with daily prospective eDiary recording.

The study will comprise four in-clinic visits and a series of structured follow-up contacts, separated by observation periods with prospective daily eDiary data collection ( Figure 2). At visit 1, eligibility will be assessed and written informed consent obtained via an electronic signature in a secure institutional digital platform (the eFORSK platform). Eligible participants will then enter a minimum 28-day baseline period, which serves both as a screening phase and to establish baseline data for endpoint assessments. A diary adherence of at least 90% (≥25 of 28 days) will be required to proceed. At visit 2, participants will be re-screened, and those meeting criteria will receive bilateral conventional greater occipital nerve (GON) blocks followed by a minimum 28-day observation period. A structured telephone call will be conducted one week after the procedure to capture any treatment-emergent adverse events and confirm scheduling for visit 3. At visit 3, eligibility will again be reassessed, including review of contraindications to CryoGON, confirmation of ≥90% diary adherence, and stable prophylactic regimens. Participants meeting criteria will undergo the CryoGON procedure (navigation-guided cryoneurolysis of the GON). Structured telephone follow-ups will be performed at weeks 1, 4, and 8, and a final in-clinic assessment will be conducted at week 12 post-CryoGON.

Considerations for woman of childbearing potential

This study will include women of childbearing potential irrespective of contraceptive use, as well as pregnant and breastfeeding women. In accordance with Article 66 of the Medical Device Regulation (MDR 2017/745), pregnant and breastfeeding participants require additional protection, including specific justification and risk minimisation measures where appropriate.

In chronic migraine, only CGRP-targeting treatments, topiramate, and onabotulinumtoxinA are supported by high-quality clinical evidence. Topiramate has recently fallen out of favour for women of childbearing potential due to the risk of neurodevelopmental disorders [12], while the safety of onabotulinumtoxinA and CGRP-inhibitors during pregnancy remains unknown, rendering them contraindicated. Consequently, effective prophylactic options are highly limited during the period when women are trying to conceive and throughout pregnancy.

Local anaesthesia administered towards the greater occipital nerve (GON) either as part of the conventional GON block or as anaesthesia as part of the CryoGON procedure, is not contraindicated in these populations, and conventional GON blocks are already in clinical use in pregnant and breastfeeding patients. ^18– 20^ Cryoneurolysis is a non-pharmacological treatment, not contraindicated in these populations, and there is no plausible rationale to suspect increased risk of harm to a nursing child or an embryo or fetus. Given the very limited alternative treatment options available, inclusion of pregnant and breastfeeding participants in this study is considered acceptable.

### Study procedure

Conventional GON-block

The conventional GON block will be performed bilaterally in an outpatient setting by trained investigators. The injection site is identified at the medial third of a line drawn between the external occipital protuberance and the mastoid process. After sterile skin preparation, a 23-gauge needle attached to a 5 mL syringe is inserted perpendicular to the skin until firm resistance is encountered, indicating contact with the periosteum. The needle is then withdrawn slightly, aspiration is performed to exclude arterial entry, and the needle is redirected superiorly.

A total of 4 mL per side of a 50:50 mixture of lidocaine 2% and bupivacaine 0.5% is injected slowly in a fan-shaped distribution to ensure adequate spread. The procedure is repeated contralaterally.

### CryoGON-procedure

The CryoGON procedure will be performed as an image-guided, percutaneous intervention. Image modalities may be per-procedural ultrasound guidance and/or surgical navigation based on MRI neurography. Devices will be used in accordance with their intended purpose and instructions for use (IFU). Study interventionists are trained surgeons with prior experience using navigation and cryoneurolysis technology and will perform the procedure according to the IFU.

For each procedure, local anesthetics will first be administered with 2–8 mL of lidocaine/adrenaline. A small skin incision of approximately 2 mm is then made at the predetermined entry point near the GON. The cryoprobe is advanced towards the target nerve under image guidance using the navigation system. Once appropriate positioning is confirmed, cryoneurolysis is applied in three cycles of 1 minute each, after which the probe is carefully retracted.

To ensure procedural consistency, the intervention will be standardised prior to study initiation. Core procedural elements, including patient positioning, target definition, cryoablation parameters, and post-procedural assessment, will be predefined. An operation form documenting all procedural elements will be completed for each intervention, and any deviations from the predefined procedure will be prospectively recorded and reported.

Participants will be observed 30 minutes post-procedure to monitor for acute adverse events and discharged home thereafter.

### Safety

Clinical experience spanning nearly half a century supports the safety of cryoneurolysis as a therapeutic approach in pain management. ^21– 23^ Known adverse device effects include injection site bleeding, hematoma and bruising. Injection site infection is reported as very rare. ^24, 25^ In contrast to other neuroablative methods, cryoneurolysis has demonstrated a low risk for neuritis, and if it occurs, it is transient. However, transient dysesthesia of a severity that affectes activities of daily living has been reported, occurring in 1,2% in one study. ^26^ Long term neuropathy induced by cryoneurolysis has never been reported. ^21, 23^ Treatment of superficial nerves can cause hyperpigmentation, depigmentation and alopecia at the treatment site, and excessively superficial lesioning can cause skin frostbite. ^23^ In five studies that targeted the occipital nerve, ^14– 16, 27, 28^ reporting from a total of 172 participants, mostly minor and transient adverse events occurred. One hematoma and 2 postprocedural occipital neurits were reported. Table 2 presents a list of known or anticipated adverse device effects associated with cryoneurolysis.

**Table 2.** Known or anticipated adverse device effects associated with cryoneurolysis. MedDRA = Medical dictionary for regulatory activities, TEAE = Treatment emergent adverse events.

MedDRA lowest level term	MedDRA code
**TEAE related to the area of administration of the CryoGON-procedure **	
Administration site infection	10066214
Administration site erythema	10074796
Administration site pain	10058049
Administration site swelling	10075107
Administration site pruritus	10075106
Administration site hyperpigmentation	10079246
Administration site hypopigmentation	10079247
Administration site bruise	10075094
Administration site haematoma	10075100
Administration site erosion	10075931
Administration site indentation	10079277
Administration site haemorrhage	10075101
**TEAE related to postprocedural disturbed occipital nerve function (i.e. not restricted to site of administration but the nerve sensory distribution area)**	
Neuropathic pain	10054095
Paraesthesia	10033775
Dysaesthesia	10013886
Hyperaesthesia	10020568
Allodynia	10053552
Hypoaesthesia	10020937
Anaesthesia	10002091

### Data collection

A predefined source data list will specify what constitutes source data in this investigation. Source documentation includes electronic patient records (EPJ) for medical history, eligibility assessments, medication use (including preventive treatments), headache diagnoses, and safety data. Data handling will comply with the General Data Protection Regulation (GDPR) and institutional requirements. Study data will be retained for 25 years after completion, as mandated by Norwegian law. Data management will follow a study-specific data management plan.

*eCRF*

An electronic Case Report Form (eCRF) will be implemented using the VieDoc™ platform (Viedoc Technologies AB), supported via the research department at St. Olav’s University Hospital. Information obtained during visits, telephone consultations, and all safety events will be entered into the eCRF. Data are stored in a pseudo-anonymised format, where participants are identified only through a unique study identification number. In case of active withdrawal, the reason (if any) will be registered in the eCRF.

*eDiary*

The eDiary application serves as the primary tool for endpoint data collection. Participants will complete daily entries throughout the baseline, GON-block, and CryoGON follow-up periods, regardless of headache status. On headache days, participants report maximum pain intensity, duration, the presence of aura, abortive medication use, and whether the headache is a migraine. A section to report alteration in participant health (e.g. potential adverse events) is available every day.

Investigators access an online dashboard that enables monitoring of diary adherence and identification of participants with insufficient entries. Entries can be made retrospectively for up to three days (current day plus the two preceding days).

*Safety data*

Safety data are collected through structured interviews at visits and telephone follow-ups, and in case of participants making contact in-between. The online dashboard presents patient-reported potential adverse events from the eDiary, which will be reviewed during study contacts. All treatment-emergent adverse events (AEs) will be registered in the eCRF.

*Questionnaires*

The Migraine-Specific Quality of Life Questionnaire v2.1(MSQ) and the Patients’ global impression of change (PGI-C) will be administered to participants. The MSQ will be collected at baseline and at week 12 following CryoGON. The PGI-C will be collected at day 1 and week 4 following conventional GON and week 8 and 12 following the CryoGON-procedure. All copyrighted instruments will be used with proper permission or license.

## Study endpoints and endpoint variables

Endpoint variable definitions follow the International Headache Society (IHS) guideline for controlled trials of preventive treatment of chronic migraine ^17^ ( Table 3). The primary endpoint variable for this study is moderate to severe headache days. Several endpoints ( Table 4) are predefined to address the two main objectives of the study, i.e. assessing whether short-term response to a conventional GON block predicts subsequent response to CryoGON and evaluating the clinical performance of the CryoGON procedure.

**Table 3.** Overview of endpoint variable definitions.

Variable	Definition
Moderate–severe headache day	A moderate/severe headache day is defined as a day with moderate or severe pain that lasts at least 4 hours or a day with a headache that is successfully treated by an acute headache medication.
CryoGON responder (≥30%; ≥50%)	≥30% or ≥50% reduction in moderate–severe headache days from baseline to weeks 9–12 after CryoGON.
GON block responder (≥30%; ≥50%)	≥30% or ≥50% reduction in moderate–severe headache days from baseline to weeks 1–4 after conventional GON block.
Self-reported migraine day	Any calendar day with ≥4 hours of headache and a participant-reported classification as a migraine, or any day with headache that is successfully treated with a triptan and/or gepant. For self-classification, participant are instructed to adhere to the following migraine day definition: A calendar day with a headache that lasts at least 4 hours; meets ICHD-III criteria C and D for migraine without aura (1.1), B and C for migraine with aura (1.2), or ICHD-III criteria for probable migraine (1.6); or a day with a headache that is successfully treated with a triptan and/or gepant.
Acute medication use	Number of days with use of acute medications per 28-day period. Analgesics used only for other purposes than headache treatment are not included.
Headache intensity	Maximum headache intensity is recorded on every day with headache at a 0-10 numerical rating scale.
MSQ v2.1	Migraine-Specific Quality of Life Questionnaire v2.1 (MSQ v2.1). The MSQ is copyrighted and registered with the United States Library of Congress by Glaxo Wellcome, Inc. MSQ v2.1 has undergone a formal translation process to Norwegian according to linguistic validation methodology. Permission for use will be obtained via Mapi Research trust before study initiation.
PGI-C	Patients’ global impression of change (PGI-C) questionnaire is public domain. PGI-C has undergone a formal translation process to Norwegian according to linguistic validation methodology.
AE	Defined according to MDR (EU 2017/745) Art. 2(57).
SAE	Defined according to MDR (EU 2017/745) Art. 2(58).
Treatment-emergent	Refers to AE that occur during or in a prespecified interval after the intervention (separately tabulated for CryoGON (occurring week 1-12 after procedure) and GON block (occurring week 1-4 after injection).
AE severity	CTCAE v6.0 grade 1-5: **Grade 1** Mild; asymptomatic or mild symptoms; clinical or diagnostic observations only; intervention not indicated. **Grade 2** Moderate; minimal, local or noninvasive intervention indicated; limiting age- appropriate instrumental ADL*. **Grade 3** Severe or medically significant but not immediately life-threatening; hospitalization or prolongation of hospitalization indicated; disabling; limiting self care ADL**. **Grade 4** Life-threatening consequences; urgent intervention indicated. **Grade 5** Death related to AE.
AE relationship assessment	The following categories from the MDCG 2020-10/1 Rev 1 will be used: **Not related:** Relationship to the CryoGON procedures can be excluded when: - the event has no temporal relationship with the procedure; - the serious adverse event does not follow a known response pattern to procedure and is biologically implausible; - the event involves a body-site or an organ that cannot be affected by the device or procedure; - the adverse event can be attributed to another cause (e.g. an underlying or concurrent illness/clinical condition, an effect of another device, drug, treatment or other risk factors. **Possible:** The relationship with the CryoGON procedures, is weak but cannot be ruled out completely. Alternative causes are also possible (e.g. an underlying or concurrent illness/clinical condition or/and an effect of another device, drug or treatment). **Probable:** The relationship with the CryoGON procedure seems relevant and/or the event cannot be reasonably explained by another cause. **Causal relationship:** the adverse event is associated with the CryoGON-procedure beyond reasonable doubt when: - the event is a known side effect of the any element of the procedure, including cryoneurolysis, or of similar devices and procedures; - the event has a temporal relationship with the procedures; - the event involves a body-site or organ that the procedure is applied to or the procedure have an effect on; - the adverse event follows a known response pattern to cryoneurolysis, - other possible causes (e.g. an underlying or concurrent illness/clinical condition or/and an effect of another device, drug or treatment) have been adequately ruled out; - harm to the subject is due to error in use Cases where relatedness cannot be assessed, or no information has been obtained will be classified as possible.
ADE	Any TEAE with possible, probable or causal relationship to the procedure.
SADE	Any TESAE with possible, probable or causal relationship to the procedure.
Procedural pain (tolerability)	NRS 0–10 discomfort during CryoGON; recorded immediately post-procedure.
Willingness to repeat (tolerability)	Participant-reported willingness to repeat CryoGON (yes/no/unsure) considering experienced benefits and drawbacks.
Technical success	Technical success is a composite (i) surgeon-confirmed completion of planned lesion at intended target with no device deficiency; (ii) Participant-reported numbness lasting >72 hours within the expected bilateral GON sensory distribution.
Device deficiency	Defined according to MDR (EU 2017/745) Art. 2(59) as any inadequacy of the medical devices used during the CryoGON procedure with respect to its identity, quality. durability, reliability, usability, safety or performance, and includes malfunctions, user errors and inadequacy in the information supplied by the manufacturer including labelling.
Procedure time	Elapsed time, measured in whole minutes, from the moment the participant enters the procedure room until the moment the participant leaves the room following completion of the CryoGON intervention.
Diary adherence	Proportion of days with completed eDiary entries per 28 day period.

GON = Greater occipital nerve, CryoGON = Cryoneurolysis of the GON, AE = Adverse event, SAE = Serious AE, ADE = Adverse device effect, SADE = Serious ADE, MDR = Medical device regulation.

**Table 4.** Overview of study endpoints.

Objective	Outcome measure	Endpoint definition	Time point
**Primary prediction endpoint**			
Evaluate whether response to a conventional GON (cGON) block predicts response to CryoGON	Change in moderate–severe headache days	Change from baseline to weeks 9–12 after CryoGON; modelled on change after GON block (weeks 1–4) as primary predictor	Baseline; weeks 1–4 (cGON); weeks 9–12 (CryoGON)
**Key secondary prediction endpoints**			
Evaluate the clinical utility of GON block as a predictor (≥30% threshold)	Dichotomised responder status (≥30%)	Proportion achieving ≥30% reduction from baseline to weeks 9–12 after CryoGON; predictor is proportion achieving ≥30% after GON-block (weeks 1–4).	Baseline; weeks 1–4 (cGON); weeks 9–12 (CryoGON)
Evaluate the diagnostic accuracy at 30% responder threshold	Sensitivity, specificity, PPV, NPV	Metrics derived from 2×2 table comparing GON-block responder (yes/no) vs CryoGON responder (yes/no) with 95% CIs.	Weeks 1–4 (cGON) and weeks 9–12 (CryoGON)
**Other secondary predication endpoints**			
Evaluate the clinical utility of cGON block as a predictor (≥50% threshold)	Dichotomised responder status (≥50% responder)	Proportion achieving ≥50% reduction from baseline to weeks 9–12 after CryoGON; predictor is proportion achieving ≥50% after GON-block (weeks 1–4).	Baseline; weeks 1–4 (cGON); weeks 9–12 (CryoGON)
Evaluate the diagnostic accuracy at 50% responder threshold	Sensitivity, specificity, PPV, NPV	Metrics derived from 2×2 table comparing GON-block responder (yes/no) vs CryoGON responder (yes/no) with 95% CIs.	Weeks 1–4 (cGON) and weeks 9–12 (CryoGON)
Evaluate the clinical utility of cGON block as a predictor (global impression of change threshold at 4 week)	Dichotomised responder status (improvement on PGI-C)	Proportion reporting improvement on PGI-C after CryoGON (week 12); predictor is the proportion reporting improvement on PGI-C after conventional GON (at week 4).	For CryoGON: At week 12 or earlier in case of study withdrawl. For cGON, at week 4
Evaluate the clinical utility of cGON block as a predictor (global impression of change threshold at day 1)	Dichotomised responder status (improvement on PGI-C)	Proportion reporting improvement on PGI-C after CryoGON (week 12); predictor is the proportion reporting improvement on PGI-C after conventional GON (at week 4 the day after procedure).	For CryoGON: At week 12 or earlier in case of study withdrawl. For cGON, at day 1
Evaluate the diagnostic accuracy, applying impression of change threshold	Sensitivity, specificity, PPV, NPV	Metrics derived from 2×2 table comparing GON-block responder (yes/no) vs CryoGON responder (yes/no) with 95% CIs.	Week 4 or day 1 (cGON), Week 12 (CryoGON)
**Primary safety endpoint**			
Evaluate the safety of CryoGON	ADEs and SADEs (device-related, treatment-emergent) number and severity	Number of SADEs Number of ADEs and proportion for each ADE with different CTCAE grad (1-5)	Procedure → Week 12
**Secondary safety endpoints**			
Evaluate the safety of CryoGON	TE-AEs and TE-SAEs	Number of TE-SAE, TE-AE with severity ≥grad 2, and TE-AE grad 1, occurring in ≥4% or more of participants	Procedure → Week 12
Evaluate the duration of numbness in the GON innervation area following CryoGON	Days with GON-sensory deficit	Average number of days with numbness	Procedure → Week 12
Evaluate the safety of Conventional GON block	TE-AEs, TE-SAEs, ADE and SADE (treatment-emergent)	Number of TE-SAE, ADE, SADE, TE-AE with severity ≥grad 2, and TE-AE grad 1 occurring in ≥4% or more of participants	Procedure → week 4
**Tolerability endpoints**			
Evaluate CryoGON procedural discomfort	NRS 0–10 score	Average maximum procedural discomfort during CryoGON procedure, recorded on an NRS (0-10) immediately post-procedure.	Procedure day
Evaluate willingness to repeat CryoGON-procedure	Willingness to repeat at week 12	Proportion answering 'yes' to willingness to repeat CryoGON considering benefits and adverse effects.	Week 12
**Primary feasibility endpoint**			
Determine the technical success	Technical success is a composite outcome(i) surgeon-confirmed completion of planned lesion at intended target with no device deficiency; (ii) participant-reported numbness >72 hours in the expected GON dermatome (bilateral) after 1 week.	Proportion of treated sides meeting both criteria	Procedure day; week 1 phone call
**Secondary feasibility endpoints**			
Evaluate the number of device deficiencies	Device deficiencies	Incidence of device deficiencies affecting the planned procedure (classified by type); per-procedure proportion with ≥1 deficiency.	Procedure day
Evaluate the diary adherence	Number of days of scheduled eDiary days with valid entries	Percentage of scheduled eDiary days with valid entries per period 28 day period; threshold ≥90% considered acceptable.	Week 1-4 post-cGON; Weeks 1–4, 5–8, 9–12 post-CryoGON
Evaluate procedure time	Procedure time in minutes from procedure initiation until completion	The average procedure time.	Procedure day
**Open label efficacy endpoints**			
Evaluate change in headache frequency	Moderate–severe headache days	Change from baseline across weeks 1–4, 5–8, and 9–12 after CryoGON.	Baseline; weeks 1–4; 5–8; 9–12
Evaluate the number of Responders applying different responder thresholds.	≥30%, ≥50%, ≥75%, 100% responder rates	Proportions meeting thresholds (primary reporting window: weeks 9–12).	Weeks 9–12 (also 1–4, 5–8)
Evaluate change in self-reported migraine days	Migraine days (self-reported)	Change from baseline to weeks 9–12 after CryoGON.	Baseline; weeks 1–4; 5–8; 9–12
Evaluate change in acute medication use	Acute medication days	Change per 28-day period across weeks 1–4, 5–8, and 9–12.	Baseline; weeks 1–4; 5–8; 9–12
Evaluate change in Quality of life	MSQ v2.1 (total & domains)	Change in MSQ from baseline to week 12.	Baseline; week 12

GON = Greater occipital nerve, cGON = Convetional GON block, CryoGON = Cryoneurolysis of the GON, AE = Adverse event, SAE = Serious AE, TE = Treatment emergent, ADE = Adverse device effect, SADE = serious ADE, PPV = Positive predictive value, NPV = Negative predictive value, NRS = Numerical rating scale, CTCAE = Common Terminology Criteria for Adverse Events.

### Endpoints related to prediction

To address the primary objective, the primary endpoint is the change in moderate–severe headache days from baseline to weeks 9–12 after CryoGON, analysed as a continuous outcome. The primary analysis estimates the association between change after the GON block (weeks 1–4; primary predictor) and change after CryoGON (weeks 9–12).

While the primary endpoint aims to evaluate whether there is an association between degree of response to conventional GON block and CryoGON treatment, this does not necessarily translate to clinical utility. A statistically significant correlation may indicate that patients who benefit more from the GON block also benefit more from CryoGON, but may not justify the use of GON block response as a binary decision-making tool.

To that end, we will estimate the predictive capability of GON block response in determining which participants will respond to CryoGON. In this analysis we will identify clinical responders, defined as a ≥30% reduction in moderate–severe headache days from baseline, in both weeks 1-4 after GON block and weeks 9–12 after CryoGON. The proportion of CryoGON responders will be compared between those who did and did not respond to GON block, estimated with a risk ratio and 95% confidence interval (CI). Additionally, the sensitivity, specificity and predictive values of response/non-response to GON block will be estimated to identify the usefulness of this treatment as a diagnostic test for response to CryoGON. These measures will also be presented with 95% CI. The 30% responder threshold is selected as it is reported to translate to a clinical meaningful change in chronic migraine. ^29^ Two additional analyses with different responder thresholds definitions are also prespecified, namely (i) 50% responder threshold and (ii) a patient-reported impression of change threshold, based on improvement on PGI-C collected at day 1 and after four weeks following cGON and at the end of study participation following CryoGON.

### Endpoints related to the open-label clinical performance of the CryoGON procedure

Open-label efficacy endpoints

The open-label efficacy of CryoGON will be evaluated based on patient-reported prospective headache diary outcomes and quality of life and patient global impression questionnaires. The migraine day endpoint variable definition is operationalized as any day with headache of minimum 4 hours duration that the participant self-identifies as a migraine, or any headache day that is successfully treated with the use of triptans and/or gepants. Patients unable to distinguish a migraine day from other headache semiologies are ineligible according to study criteria. As such, the ability of participant to identify a migraine day is part of the review at the time of inclusion and ensures that the endpoint is operationalized in a manner that adheres to the HIS’ guideline. ^17^ Change in quality of life will be measured with the MSQ v2.1 questionnaire at baseline and week 12. Patient global impression of change will be collected at week 8 and week 12.

Open label safety, tolerability, and feasibility endpoints

Safety will be assessed separately for CryoGON (weeks 1-12 following the CryoGON procedure) and conventional GON block (weeks 1-4 following conventional GON injection) as treatment-emergent adverse events (TEAEs) and treatment emergent serious adverse events (TESAEs), adverse device effects (ADEs) and serious ADE (SADEs). Treatment-emergent refers to all AEs events at or following initiation of the conventional GON (week 1-4) or CryoGON procedure (week 1-12), respectively. All AEs will be coded according to the Medical dictionary for regulatory activities (MedDRA) and grouped and summarised using the lowest level term (LLTs).

Severity assessment will apply the standardized grading criteria from the Common Terminology Criteria for Adverse Events (CTCAE) version 6. Casualty assessment will follow the definition of the Medical Device Coordination Group, ^30^ and all TEAE will be categorized as not related or possible, probable or casual related to conventional GON in week 1-4 post-procedure and the CryoGON-procedure in week 1-12 post-procedure. An adverse device effects (ADE) is defined according to ISO 14155:2020 as any adverse event related to the use of an investigational device. This is operationalised for this study as any AE that is assessed to have possible, probable or causal relationship to the CryoGON procedure.

Tolerability will primarily be evaluated by intra-procedural discomfort (NRS 0–10) and additionally, the willingness of the participant to repeat the CryoGON procedure. At week 12, or earlier in the event of participant withdrawal, participants will be explicitly asked to consider the balance of perceived benefits and drawbacks of CryoGON and respond with ‘yes’, ‘no’, or ‘unsure’ regarding willingness to undergo the procedure again. The primary feasibility endpoint will be technical success rate. Technical success is defined as a composite of (i) surgeon-confirmed completion of the planned cryo-lesion at the intended target without device deficiency, and (ii) participant-reported numbness persisting >72 hours in the GON peripheral innervation area as reported by the participant on a telephone call after one week. The second criterium is included with the intention to distinguish prolonged sensory change consistent with cryoneurolysis from transient anaesthesia due to local anaesthetic used during the procedure. Other feasibility endpoints are number of device deficiencies, CryoGON procedure duration and diary adherence across study periods.

Adequate diary adherence is essential for the valid estimation of headache day–based endpoints, particularly responder rates. Therefore, diary adherence is included as an operational feasibility endpoint to enable evaluation of the robustness of the data underpinning both the primary endpoint and the open-label efficacy analyses.

## Statistical analysis

This trial is designed to evaluate if and to what degree response to conventional GON blocks predict response to CryoGON-treatment. For the primary endpoint the following hypothesis is testet:

• **Null hypothesis (H _0_):** There is no relationship between the degree of treatment response to conventional GON block and CryoGON treatment (regression slope (β _1_) = 0)
• **Alternative hypothesis (H _1_):** There is an association between the degree of treatment response to conventional GON block and CryoGON treatment (regression slope (β _1_) ≠ 0)

### Protocol violations

Protocol violations in this study are headache diary nonadherence defined as <90% adherence and/or alterations in headache preventive treatment during trial participation. Electronic headache diary adherence will be calculated at 4-week (28 days) intervals.

### Statistical analysis

The main analysis is planned when all participants have concluded the 12-week follow-up after the CryoGON procedure and the database has been locked. A statistical analysis plan (SAP) will be finalized prior to data lock.

The full analysis set (FAS) will include all participants that received CryoGON-treatment to adhere to the intention to treat ideal and will be used for the main analysis. Two additional populations are defined for the purpose of sensitivity analyses. The per protocol set (PPS) includes participants without protocol violations. A technical success set (TSS) includes the subset of participants that fulfil the criteria for a technical successful CryoGON-treatment.

The analysis of the primary endpoint will use linear regression, with change after CryoGON as the dependent variable and change after the GON block (measured during weeks 1–4) as the main predictor. For participants who do not complete the eDiary on all days, the number of moderate-severe headache days will be calculated based on the available data and estimated for a 28-day period before calculating the change in number of moderate-severe headache days.

The key secondary outcome is defined as a ≥30% reduction in moderate to severe headache days from baseline to weeks 9–12 after CryoGON, treated as a binary response (yes/no). The corresponding GON block response is also defined dichotomously using the same threshold, assessed during weeks 1–4. The proportion of participants with ≥30% response to CryoGON will be compared between those who did and did not respond to GON block using a risk ratio with 95% confidence interval (CI).

Additional diagnostic performance measures—sensitivity, specificity, positive predictive value (PPV), and negative predictive value (NPV)—will also be calculated with 95% CI.

As this is an exploratory pilot study and the study has one specified primary endpoint, no adjustment of multiplicity is planned.

### Sensitivity analyses

We prespecify a key sensitivity analysis restricted to the TSS population to estimate effect sizes conditional on technical successful CryoGON delivery as per prespecified criteria. We highlight this analysis because it reflects the expected performance of CryoGON when delivered as intended and might better mirror real world practice, where an unsuccessful first attempt would typically prompt retreatment rather than be taken as evidence of inefficacy. To preserve interpretability and precision of this key sensitivity analysis, recruitment will continue until the TSS population reaches the sample size specified by the power calculation.

Additional sensitivity analysis

• Restricted analysis to the per protocol population.
• Adjusted analysis to account for cyclical variation in headache frequency related to injection-based preventives (e.g., onabotulinumtoxinA, CGRP monoclonal antibodies). Analysis adjusting for other variables, such as treatment expectation, sex and age will be prespecified in the SAP.

### Subgroup analysis

Subgroup analyses will be prespecified in the SAP, and may include exploring differences in effect according to the degree of numbness following CryoGON (hypoaesthesia vs anaesthesia) and comparing bilateral versus unilateral successful CryoGON treatment.

### Sample size

The primary scientific question is whether the response to the GON block can predict treatment outcome after CryoGON. This will be assessed both in terms of whether there is a linear association between the degree of response to GON block and response to CryoGON (primary endpoint), and whether a ≥30% response to GON block is associated a greater chance of response to CryoGON and can be used as a diagnostic test to identify those who are likely to respond to CryoGON (key secondary endpoints). Both analysis strategies are considered in the sample size calculation, together with an assessment of what are feasible recruitment numbers. Ultimately, the target samples size was set to 70 based on feasibility and the importance of the precision of estimates.

The primary analysis tests the association between change in moderate-to-severe headache days after the GON block (weeks 1–4) and change after CryoGON (weeks 9–12) using linear regression (with the null hypothesis that there is no association between the response to GON block and CryoGON, i.e. β _1_ = 0). The proposed advantage of CryoGON is a much longer duration of treatment effect than is observed with conventional GON block. Thus, an association of 0.5 will be considered clinically meaningful, that is to say the gradient of the regression slope is 0.5, and thus for each 1-day reduction in moderate-severe headache days in weeks 1-4 after GON block, the average reduction is half a day in week 9-12 following the CryoGON-procedure. Assuming a standard deviation 5 for change in number of moderate-severe headache days from baseline, 26 participants will be sufficient to provide 80% power to detect a regression slope of 0.5 at 5% significance level.

The key secondary outcomes include a comparison of the proportion of responders to CryoGON (≥30% reduction in moderate-severe headache days) among those with and without response to GON block. This will be reported as a risk ratio (95% CI), along with sensitivity, specificity, PPV and NPV (also with 95% CI). We expect that around 40% will respond to GON block based on results from Chowdhury et al. (2021), ^5^ who reported ~20% response using a 50% threshold. Among those who do not respond to GON block, we anticipate that around 30% will respond to CryoGON. For GON block to provide reasonable predictive information, the response to CryoGON among those who respond to GON block should be around 80% (i.e. a 50-percentage point increase in response). This would also equate to a sensitivity of 64% and specificity of 80%. A sample size of 31 (18 in the GON block negative and 13 in the GON block positive group) will be sufficient to achieve 80% power to detect a 50-percentage point difference (and risk ratio of 2.67) at 5% significance level and using a Chi-squared test. However, this would provide imprecise estimates of the sensitivity and specificity of GON block. With a target sample size of 70, we will have sufficient data to estimate a 95% CI around the sensitivity and specificity of a width of approximately 17 and 12 percentage points, respectively.

### Study monitoring

The CryoGON trial will be monitored by independent clinical research monitors in accordance with a dedicated monitoring plan. The plan will be risk-adapted and developed on the basis of a project-specific risk analysis. Monitoring will focus on data integrity, adherence to protocol, regulatory compliance, and participant safety.

An independent Data Safety Monitoring Committee (DSMC) will be established. The DSMC will review accumulating safety data after approximately 25% and 50% of participants have undergone the CryoGON procedure, and will provide recommendations to the sponsor regarding continuation, modification, or termination of the trial based on the observed safety profile.

### Data access

At the time of database lock following study completion, only the study statistician will be granted full access to the dataset for the purpose of conducting the analyses prespecified in the Statistical Analysis Plan (SAP). After the preplanned analyses are finalised, the full pseudonymised dataset will be made available to all investigators involved in the study, enabling independent verification of results and preparation of publications. Data sharing will be governed by applicable data protection legislation and no contractual restrictions will apply.

## Discussion

The present study protocol outlines a prospective clinical investigation of the CryoGON procedure for the preventive treatment of chronic migraine. Conventional local anaesthetic GON blocks are already in use for transitional or acute treatment in headache care, but their short duration of effect limits their role as a preventive strategy. Cryoneurolysis, by inducing a reversible long-acting nerve block through controlled axonotmesis, offers the possibility of a more sustained relief. If effective, this approach may open an entirely new non-pharmacological treatment avenue for chronic migraine, a patient group often in need of additional options.

This trial makes two key contributions. First, it will generate the largest open-label dataset to date on safety, tolerability, feasibility, and efficacy of CryoGON in chronic migraine. These data will inform the design of future studies, including the feasibility and justification of a sham-controlled trial. Second, it will evaluate the predictive value of a conventional GON block in identifying likely CryoGON responders. The primary endpoint assesses the continuous relationship between reduction in headache days following the GON block and after CryoGON, thereby addressing the scientific question of whether the two responses are correlated. The key secondary endpoint complements this by estimating the diagnostic performance of a dichotomised response threshold (≥30% reduction), reporting sensitivity, specificity, positive and negative predictive values. Together, these endpoints provide clinically applicable evidence that can suggest whether GON block response can serve as a decision-making tool for selecting patients for CryoGON treatment.

The study is conducted within a research environment with long-standing expertise in investigator-initiated interventional trials in headache disorders. The infrastructure for recruitment, eDiary-based outcome collection, and rigorous monitoring ensures both high level of data quality and study feasibility. User involvement has been central from the outset. Patient representatives from the Norwegian patient organization contributed to the decision to prioritise chronic migraine for this trial and remain engaged in its planning and implementation. This aligns the study with patient priorities and strengthens its potential for clinical translation.

The absence of a sham-controlled comparator is a key limitation of the present study. As both conventional GON block and CryoGON are invasive interventions, non-specific effects such as treatment expectation, contextual effects, and individual response propensity may contribute to observed improvements and to any association between responses to the two treatments. Consequently, an observed relationship between response to GON block and response to CryoGON cannot disentangle shared physiological mechanisms from shared expectancy-related effects, nor does it establish that response to a GON block causally predicts response to CryoGON at the individual level. To partially address this, analyses adjusting for baseline treatment expectation will be prespecified. Overall, findings related to the predictive utility of GON block response should be interpreted as hypothesis-generating and primarily as informing feasibility, effect size estimates, and the rationale for subsequent sham-controlled trials.

## Other required information

## Ethics approval

Appropriate approvals will be obtained prior to study initiation.

## Registration

The trial will be registered in clinical trials.gov before study initiation.

## Data Availability

No data is associated with this stage 1 protocol publication. This project contains the following extended data: (Wergeland, Tore (2025)) Spirit checklist: https://doi.org/10.6084/m9.figshare.30355048. ^31^ Data are available under the terms of the Creative Commons Zero “No rights reserved” data waiver (CC0 1.0 Public domain dedication).
