# Supplementary material for: Stage 1 Registered Report:  Rationale, design and study protocol for a prospective, exploratory study Predicting Response to image-Guided Cryoneurolysis of the Greater Occipital Nerve (CryoGON) in Chronic Migraine Using a Conventional Greater Occipital Nerve Block
Source: F1000Res. 2026 Feb 4;14:1157. Originally published 2025 Oct 22. [Version 2] doi: 10.12688/f1000research.171975.2 (PMC13309775; doi:10.12688/f1000research.171975.2)
Supplement: Peer review history

# Reviewer report: Reviewer response for version 2

**DOI:** 10.5256/f1000research.196068.r488434

**Published:** 2026-06-26

**Authors:** Dagmareier Beier

**Affiliations:** 1 Odense University Hospital, Odense C, Denmark

The authors plan to perform a prospective, single-site open-label, exploratory study evaluating whether response to a conventional GON block predicts subsequent response to CryoGON and assessing open-label safety, tolaerability, feasibility and efficacy of CryoGON.

 The rational of the study is described clearly and the design appropriate.

 However, there are some issues to be adressed:

 1) Patients with MOH are not excluded from the trial. This may affect the treatment efficacy, when patients responsive to the treatment reduce their intake of pain killers.

 2) Patients with other primary headache disorders are not excluded. This may affect the experienced treatment efficacy.

 3) The study is not placebo controlled, which may affect the outcome as well.

Is the study design appropriate for the research question?

Yes

Is the rationale for, and objectives of, the study clearly described?

Yes

Are sufficient details of the methods provided to allow replication by others?

Yes

Are the datasets clearly presented in a useable and accessible format?

Yes

Reviewer Expertise:

headache disorders, neurology

I confirm that I have read this submission and believe that I have an appropriate level of expertise to confirm that it is of an acceptable scientific standard.

# Reviewer report: Reviewer response for version 2

**DOI:** 10.5256/f1000research.196068.r486407

**Published:** 2026-06-19

**Authors:** Faraidoon Haghdoost

**Affiliations:** 1 The George Institute for Global Health, Sydney, New South Wales, Australia

Thank you for the opportunity to review this protocol. The study addresses an interesting and clinically relevant question regarding the potential role of conventional greater occipital nerve (GON) block response in predicting outcomes following image-guided cryoneurolysis of the greater occipital nerve (CryoGON) in chronic migraine.

 The manuscript is generally well written, and the protocol is clearly described. I have several suggestions that may improve the clarity and rationale of the manuscript.

**Title**

 The title is somewhat difficult to follow and could be simplified to improve readability. Consider restructuring it to make the study objective clearer. Possible alternatives include:

 * *Predicting Response to Image-Guided Cryoneurolysis of the Greater Occipital Nerve (CryoGON) in Chronic Migraine Following a Conventional Greater Occipital Nerve Block: Rationale, Design, and Study Protocol for a Prospective Exploratory Study*

 * *Rationale, Design, and Study Protocol for a Prospective Exploratory Study of Predicting Response to Image-Guided Cryoneurolysis of the Greater Occipital Nerve (CryoGON) in Chronic Migraine Following a Conventional Greater Occipital Nerve Block*

**Abstract**

 I suggest reporting the planned sample size in the abstract, as this is an important study design characteristic and helps readers quickly understand the scale of the study.

 Introduction

 The statement:

 "Migraine is a disabling primary headache disorder with a lifetime prevalence of 17% of the population and ranking as the second leading cause of disability in females under 50 years of age."

 may not accurately reflect the current Global Burden of Disease literature. Migraine is generally considered the leading cause of disability among young women and the second leading cause of disability worldwide among all causes. I recommend revising this statement to better align with the current evidence. 

 Please ensure that all abbreviations are defined at first use, including commonly used terms such as CGRP. In addition, some abbreviations appear to be defined multiple times throughout the manuscript. For example, GON is introduced on several occasions. Defining abbreviations once and then using them consistently throughout the manuscript would improve readability.

 For the sentence:

 "A recent randomized trial and a meta-analysis support modest efficacy in chronic migraine, with reductions in headache days and responder rates superior to placebo."

 I suggest clarifying the intervention being discussed. For example:

 "A recent randomised trial and a meta-analysis support modest efficacy of GON interventions in chronic migraine, with reductions in headache days and responder rates superior to placebo."

 Most importantly, the rationale for the study requires further clarification. Based on the introduction, CryoGON appears to remain an emerging intervention in primary headache disorders, with limited evidence and ongoing studies evaluating its efficacy in migraine. Therefore, it is not entirely clear why prediction of response to CryoGON based on response to a conventional GON block is being investigated at this stage particularly when evidence supporting the efficacy of CryoGON itself in migraine remains limited.

 Specifically, it would be helpful for the authors to clarify:

 * Whether sufficient evidence exists to support the efficacy of CryoGON in chronic migraine.

 * Whether there is existing evidence suggesting that response to a conventional GON block predicts response to CryoGON.

 * The biological or clinical rationale linking response to these two procedures.

 A more detailed explanation of the rationale underpinning this predictive study would strengthen the introduction and help readers understand the importance of the research question.

**Methods**

 The protocol would benefit from explicitly describing the contraindications to CryoGON. Including these would improve completeness and help readers better understand participant selection and safety considerations.

 I would also suggest rephrasing the following sentence:

 "This study will include women of childbearing potential irrespective of contraceptive use, as well as pregnant and breastfeeding women."

 As currently written, the sentence may inadvertently suggest that the study population is limited to these groups. Consider rewording it to clarify that these participants are eligible for inclusion alongside all other eligible participants.

 For patient-reported outcome measures such as the Migraine-Specific Quality of Life Questionnaire version 2.1 (MSQ) and the Patient Global Impression of Change (PGI-C), it would be helpful to specify whether only English-language versions will be used or whether validated translated versions are available. Including this information would improve reproducibility and provide readers with a clearer understanding of how patient-reported outcomes will be collected.

Is the study design appropriate for the research question?

Yes

Is the rationale for, and objectives of, the study clearly described?

Partly

Are sufficient details of the methods provided to allow replication by others?

No

Are the datasets clearly presented in a useable and accessible format?

Yes

Reviewer Expertise:

Migraine and headache disorders

I confirm that I have read this submission and believe that I have an appropriate level of expertise to confirm that it is of an acceptable scientific standard, however I have significant reservations, as outlined above.

## References

[1] : Migraine remains second among the world’s causes of disability, and first among young women: findings from GBD2019. *The Journal of Headache and Pain* .2020;21(1) : 10.1186/s10194-020-01208-0 10.1186/s10194-020-01208-0 33267788 PMC7708887

# Reviewer report: Reviewer response for version 2

**DOI:** 10.5256/f1000research.196068.r458946

**Published:** 2026-02-27

**Authors:** Nourhan Abdelmohsen Taha

**Affiliations:** 1 Ain Shams University, Cairo, Cairo Governorate, Egypt

Peripheral nerve blocks especially, greater occipital nerve block has been widely used specially in  middle and low income countries for it  feasibility and cost-effectivenss, yet the short term efficiay of the landmark guided GON block is still a limitation, thus providing a long lasting reversible nerve block modality is a new hope to chronic migraine patients.

 In my opinion the methodology is well described and planned to fulfill the aim of the study, yet I have a consideration as regards the difference in localization technique of GON for each treatment modality, that would limit the comparability of the responders rate 

 The localization technique used in cryo group is imaging guided that increases the probability of good response, so I prefer if the conventional group be injected ultrasound guided, to elleviate this limitaion in results comparability.

Is the study design appropriate for the research question?

Yes

Is the rationale for, and objectives of, the study clearly described?

Yes

Are sufficient details of the methods provided to allow replication by others?

Yes

Are the datasets clearly presented in a useable and accessible format?

Yes

Reviewer Expertise:

headache disorders, peripheral nerve blocks

I confirm that I have read this submission and believe that I have an appropriate level of expertise to confirm that it is of an acceptable scientific standard, however I have significant reservations, as outlined above.

# Reviewer report: Reviewer response for version 1

**DOI:** 10.5256/f1000research.189650.r441857

**Published:** 2026-01-08

**Authors:** Gulcin Babaoglu

**Affiliations:** 1 Ankara Bilkent City Hospital, Ankara, Turkey

This protocol addresses a clinically meaningful and innovative question by investigating whether a conventional greater occipital nerve (GON) block can be used as a potential clinical guidance tool for image-guided cryoneurolysis (CryoGON) in patients with chronic migraine. The prospective design, use of electronic diaries (eDiary), and a prespecified analysis plan are notable strengths of the study.

 However, in its current form, several aspects related to patient recruitment, interpretation of the predictive framework, statistical evaluation, and interventional standardization would benefit from further clarification in order to enhance the scientific robustness and feasibility of the study.

 1. When the inclusion and exclusion criteria are considered together, a situation arises that requires clarification regarding the inclusion of patients receiving onabotulinumtoxinA (Botox) treatment.

 Inclusion Criterion 5 requires that patients using Botox must have completed at least three consecutive PREEMPT injection cycles and be on a stable regimen.

 Exclusion Criterion 1 excludes patients who have received injections to the occipital or suboccipital region within the last 12 weeks prior to baseline.

 The PREEMPT protocol anatomically includes injections to the occipital region and is administered at 12-week intervals.

 Inclusion Criterion 6 requires that no changes be made in the dose or frequency of preventive treatments.

 Assessment:

 The concurrent application of these criteria appears likely to create practical difficulties in enrolling patients who are on stable Botox treatment. Even in cases where patients are considered non-responders after three injection cycles, this situation needs to be explicitly stated in the protocol. In summary, regardless of response status, patients receiving PREEMPT treatment may overlap with the exclusion window.

 2. The primary objective of the study is to investigate whether the response to a conventional GON block is associated with the response following CryoGON, and thereby to assess whether the GON block can serve as a potential clinical guidance tool for CryoGON.

 However, the open-label design of the study and the invasive nature of both interventions necessitate a cautious interpretative framework when evaluating the findings. An observed association between GON block response and CryoGON response may reflect a shared physiological mechanism; however, it may also reflect patients’ general tendency to respond to interventional treatments, treatment expectation, or individual response characteristics.

 In this context, the continuous response relationship evaluated using linear regression essentially demonstrates an association between the responses to the two treatments. The use of this association as a clinical prediction or decision-making tool is limited to the prespecified dichotomous response analyses and remains exploratory in nature.

 In summary, patients with a strong placebo response are more likely to respond to both interventions (“placebo consistency” or expectation effect).

 3. The CryoGON procedure is described in the protocol in a generally clear manner and provides a sufficient foundation with respect to clinical applicability and safety of the intervention. However, within the context of a protocol publication, a more explicit description of certain technical details related to interventional standardization and reproducibility across different centers could strengthen the methodological rigor of the study.

 In particular, referencing only the instructions for use (IFU) may create ambiguity regarding which aspects of the procedure are fixed and which may vary depending on the operator or clinical conditions.

 4. In the secondary analyses, it is planned to dichotomize the response to the GON block using a fixed threshold such as ≥30% and to calculate sensitivity and specificity.

 While this approach is clinically intuitive, categorizing a continuous variable based on a single threshold may result in loss of information and may not fully reflect the predictive performance of the GON block across all possible thresholds.

Is the study design appropriate for the research question?

Partly

Is the rationale for, and objectives of, the study clearly described?

Yes

Are sufficient details of the methods provided to allow replication by others?

Partly

Are the datasets clearly presented in a useable and accessible format?

Partly

Reviewer Expertise:

pain medicine

I confirm that I have read this submission and believe that I have an appropriate level of expertise to confirm that it is of an acceptable scientific standard, however I have significant reservations, as outlined above.

# Response

**Published:** 2026-02-01

**Authors:** Tore Wergeland

**Affiliations:** Norwegian University of Science and Technology Department of Neuromedicine and Movement Science, Trondheim, Trøndelag, Norway

*Thank you for your comments. We address them in the following:*

 1. When the inclusion and exclusion criteria are considered together, a situation arises that requires clarification regarding the inclusion of patients receiving onabotulinumtoxinA (Botox) treatment.

 Inclusion Criterion 5 requires that patients using Botox must have completed at least three consecutive PREEMPT injection cycles and be on a stable regimen.

 Exclusion Criterion 1 excludes patients who have received injections to the occipital or suboccipital region within the last 12 weeks prior to baseline.

 The PREEMPT protocol anatomically includes injections to the occipital region and is administered at 12-week intervals.

 Inclusion Criterion 6 requires that no changes be made in the dose or frequency of preventive treatments.

 Assessment:

 The concurrent application of these criteria appears likely to create practical difficulties in enrolling patients who are on stable Botox treatment. Even in cases where patients are considered non-responders after three injection cycles, this situation needs to be explicitly stated in the protocol. In summary, regardless of response status, patients receiving PREEMPT treatment may overlap with the exclusion window.

***Response:***

*You are correct, and we thank the reviewer for identifying this inconsistency. Exclusion criterion 1 was intended to exclude other occipital or suboccipital injections, but not onabotulinumtoxinA administered according to the PREEMPT protocol. This has now been explicitly clarified in the exclusion criteria. Patients receiving PREEMPT treatment are eligible provided they are on a stable regimen and meet all other inclusion criteria, irrespective of response status.*

 2. The primary objective of the study is to investigate whether the response to a conventional GON block is associated with the response following CryoGON, and thereby to assess whether the GON block can serve as a potential clinical guidance tool for CryoGON.

 However, the open-label design of the study and the invasive nature of both interventions necessitate a cautious interpretative framework when evaluating the findings. An observed association between GON block response and CryoGON response may reflect a shared physiological mechanism; however, it may also reflect patients’ general tendency to respond to interventional treatments, treatment expectation, or individual response characteristics.

 In this context, the continuous response relationship evaluated using linear regression essentially demonstrates an association between the responses to the two treatments. The use of this association as a clinical prediction or decision-making tool is limited to the prespecified dichotomous response analyses and remains exploratory in nature.

 In summary, patients with a strong placebo response are more likely to respond to both interventions (“placebo consistency” or expectation effect).

***Response:** We agree with the reviewer that, given the open-label design and the invasive nature of both interventions, any observed association between response to conventional GON block and CryoGON must be interpreted with caution. An observed relationship may reflect shared physiological mechanisms, but may also reflect non-specific factors such as treatment expectation or individual response propensity (“placebo consistency”).*

*Accordingly, the primary analysis is explicitly framed as an assessment of association rather than causal prediction. The dichotomised responder analyses are intended to explore potential clinical utility in a hypothesis-generating manner, and not to establish a definitive decision rule. This limitation has been clarified in the Discussion section of the manuscript.*

 3. The CryoGON procedure is described in the protocol in a generally clear manner and provides a sufficient foundation with respect to clinical applicability and safety of the intervention. However, within the context of a protocol publication, a more explicit description of certain technical details related to interventional standardization and reproducibility across different centers could strengthen the methodological rigor of the study.

 In particular, referencing only the instructions for use (IFU) may create ambiguity regarding which aspects of the procedure are fixed and which may vary depending on the operator or clinical conditions.

***Response: **We have now clarified that core procedural elements will be standardized prior to study initiation in the manuscript. An operation form capturing predefined procedural parameters will be completed for each procedure, and any deviations will be documented and reported. This approach is intended to enhance reproducibility while allowing documentation of clinically necessary variations.*

 4. In the secondary analyses, it is planned to dichotomize the response to the GON block using a fixed threshold such as ≥30% and to calculate sensitivity and specificity.

 While this approach is clinically intuitive, categorizing a continuous variable based on a single threshold may result in loss of information and may not fully reflect the predictive performance of the GON block across all possible thresholds

***Response:** We agree that dichotomising a continuous outcome may result in loss of information. This is why the primary analysis evaluates the association between responses to the two interventions as continuous variables using linear regression. The dichotomised responder analyses (30%, 50%, and PGI-C–based thresholds) are prespecified secondary analyses intended to explore potential clinical applicability across commonly used responder definitions.*

# Reviewer report: Reviewer response for version 1

**DOI:** 10.5256/f1000research.189650.r434098

**Published:** 2025-12-11

**Authors:** Nathaniel M. Schuster

**Affiliations:** 1 University of California, San Diego, CA, USA

Thank you for inviting me to review this protocol for a planned open-label prospective single-arm study evaluating whether landmark-based occipital nerve block predicts response to image-guided cryoneurolysis. It is overall well-written with some minor grammatical issues. Design-wise, I would want to know whether response to greater occipital nerve block over the first 6 hours (duration of local anesthetic) or whether response at 4 weeks better predicts response to cryo. I would also want to know whether greater occipital nerve blocks predict response to cryoneurolysis at 3 months- 4 weeks as proposed is too short.

Is the study design appropriate for the research question?

Yes

Is the rationale for, and objectives of, the study clearly described?

Yes

Are sufficient details of the methods provided to allow replication by others?

Yes

Are the datasets clearly presented in a useable and accessible format?

Not applicable

Reviewer Expertise:

Headache, interventional pain, cannabinoids, neuromodulation

I confirm that I have read this submission and believe that I have an appropriate level of expertise to confirm that it is of an acceptable scientific standard, however I have significant reservations, as outlined above.

# Response

**Published:** 2026-02-01

**Authors:** Tore Wergeland

**Affiliations:** Norwegian University of Science and Technology Department of Neuromedicine and Movement Science, Trondheim, Trøndelag, Norway

Thank you for your thoughtful comments.

 We agree with the reviewer that both the short-term abortive effect of a conventional greater occipital nerve (GON) block, in addition to its longer-term impact on headache frequency, could be scientifically and clinically informative when evaluating its predictive value for CryoGON outcomes.

 However, in the current study, treatment response is defined as the change in moderate-to-severe headache days derived from prospective headache diaries, reflecting a preventive rather than abortive treatment framework. Because participants may receive the conventional GON block on headache-free days, the study design does not permit a systematic assessment of short-term abortive effects within the first several hours following injection.

 To partially address short-term effects of a conventional GON block, we have added collection of the Patient Global Impression of Improvement (PGI-I) on day 1 (the day following the conventional block procedure). The manuscript has been updated to include this as an endpoint. In addition, we will document the presence or absence of headache at the time of the procedure and, when applicable, the participant-reported duration of headache following the injection. These data may be explored in post hoc analyses, if deemed feasible.

 Extending the observation period following the conventional GON block to 12 weeks in order to assess longer-term predictive effects was considered. However, this would substantially prolong study participation and require prolonged stability of preventive treatments, which was not deemed feasible within the current design. The present study therefore focuses on the association between short-term changes in headache frequency following GON block (weeks 1–4) and subsequent response to CryoGON (weeks 9–12), with the aim of generating foundational data to inform the design of future studies specifically addressing these clinically relevant timing questions.
